# Supplementary material for: Regulation of Cyclooxygenase-2 Expression in Human T Cells by Glucocorticoid Receptor-Mediated Transrepression of Nuclear Factor of Activated T Cells
Source: Int J Mol Sci. 2022 Oct 31;23(21):13275. doi: 10.3390/ijms232113275 (PMC9653600; doi:10.3390/ijms232113275)
Supplement: Supplementary file 1 [file ijms-23-13275-s001.zip › ijms-1869201-supplementary.pdf]

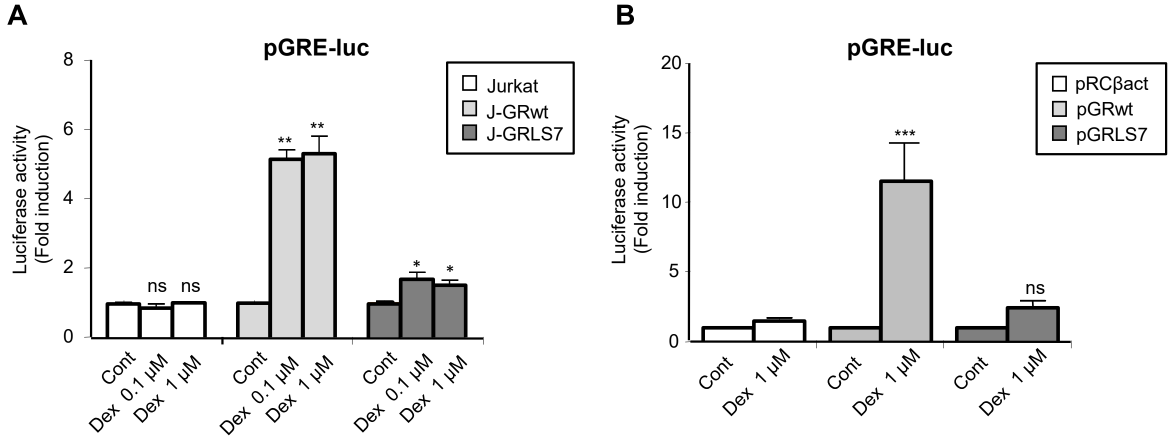

Figure S1. Dex mediated transactivation through a GRE is dependent on GR binding to DNA. **A**) Parental, J–GRwt and JGRLS7 Jurkat cells were transiently transfected with a luciferase reporter construct containing a GC response element (pGRE−Luc). **B**) Parental Jurkat cells were cotransfected with GR expression plasmids (pGRwt and pGRLS7) or with the empty vector pRCβact, along with the reporter plasmid pGRE−Luc. Cells were treated for 18 h, with Dex (0.1 to 1 μM). Luciferase activity is represented as fold induction over unstimulated cells (mean ± SEM). ns: non significant; *p<0.05, **p<0.01; ***p<0.001 Dex treatments vs Cont). Results shown are from a representative of three independent experiments performed in triplicate

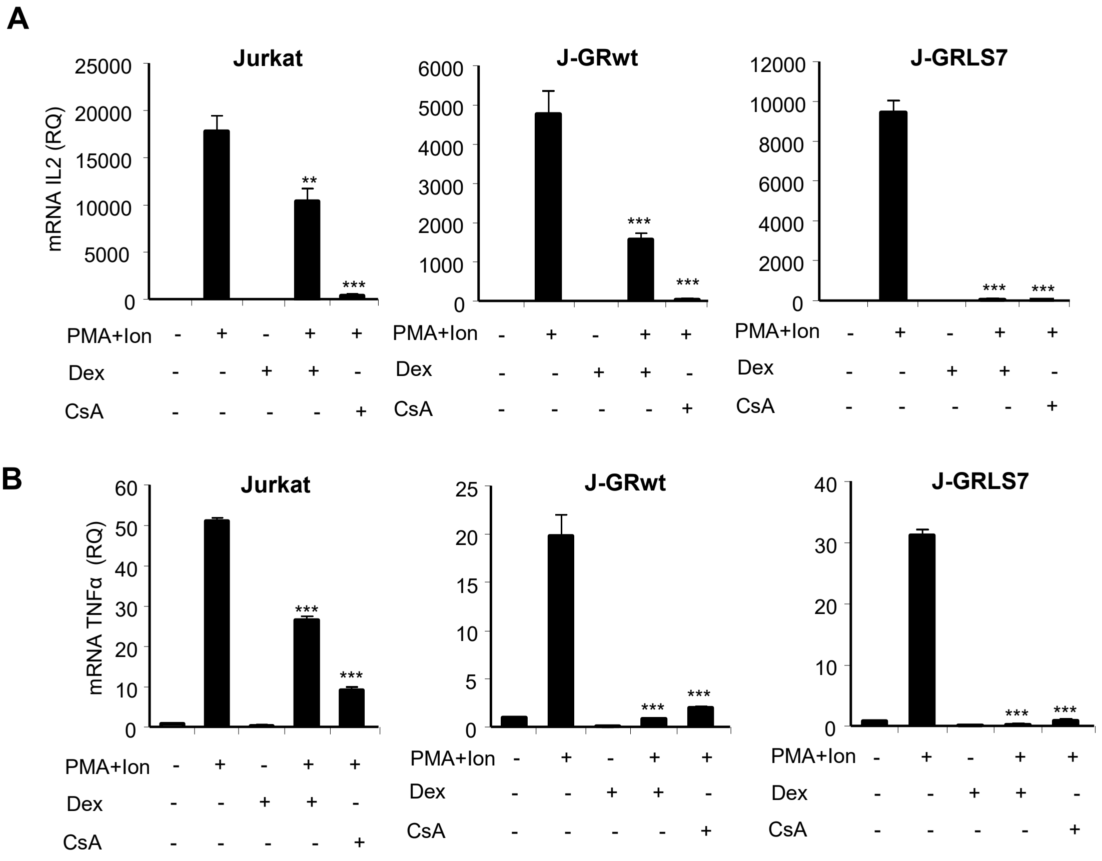

Figure S2. Effect of Dex on IL2 and TNFα expression in activated Jurkat cells. Analysis of IL2 **(A)** and TNFα **(B)** mRNA levels by quantitative real-time RT–PCR in Parental, J–GRwt and J–GRLS7 Jurkat cells treated with PMA+Ion (15 ng/ml +1 µM) for 18h in the presence or absence of Dex (1 µM) or CsA (100 ng/ml). Results are shown as RQ ± SEM (**p<0.01 *** p<0.001 vs PMA+Ion treatment). Results shown are from a representative of two independent experiments performed in triplicate.

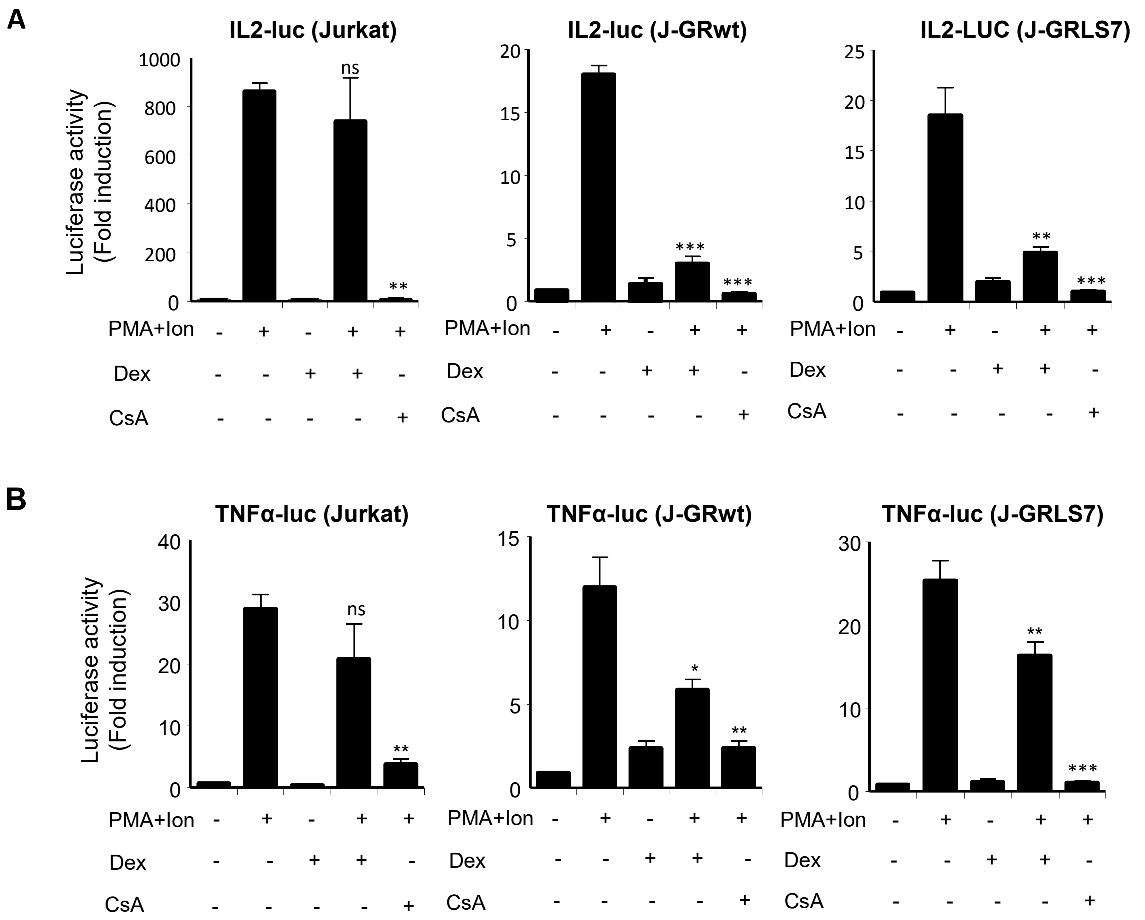

Figure S3. Analysis of Dex effects on IL2 and TNFα promoter activities. Parental, J–GRwt and J–GRLS7 Jurkat cells were transfected with luciferase reporter constructs bearing IL2 **(A)** or TNFα **(B)** promoters. Cells were cultured in the absence (Cont) or presence of PMA + Ion for 16 h and assayed for luciferase activity. Dex (1 µM) or CsA (100 ng/ml) were added 1 h before stimulation. Results are represented as fold induction of RLUs in PMA+Ion samples over unstimulated control ones (mean ± SEM). ns: non significant; *p<0.05, **p<0.01; ***p<0.001 vs PMA+Ion). Results shown are from a representative of three independent experiments performed in triplicate.
